# Integrating replication kinetics and ultrastructural analysis to identify targets for optimizing rVSV bioproduction

**DOI:** 10.1128/spectrum.01063-25

**Published:** 2026-03-24

**Authors:** Rebecca Habisch, Johannes G. Wieland, Jorge Soza-Ried, Clarissa Read, Paul Walther, Peter Neubauer, Eva Puschmann, Martin Dass

**Affiliations:** 1 Boehringer Ingelheim, Viral Therapeutics Center, Ochsenhausen, Germany; 2 Central Facility Electron Microscopy, Ulm University 9189, Ulm, Germany; 3 Technische Universität Berlin, Institute of Biotechnology, Chair of Bioprocess Engineering 672110 , Berlin, Germany; Barnard College, Columbia University, New York, New York, USA

**Keywords:** therapeutic virus production, virus replication kinetics, transmission electron microscopy, vesicular stomatitis virus, rVSV

## Abstract

The growing demand of viral products for gene therapy and as oncolytic agents underscores the importance of optimizing virus production processes in the biopharmaceutical industry. Beyond established bioprocess parameters such as infectious titer, additional analytical methods are required to gain deeper insights into these processes. Electron microscopy, a well-established technique in virology, is rarely utilized in bioprocess design. In this study, we aim to identify potential targets for bioprocess optimization by combining ultrastructural changes of infected producer cells with the replication kinetics of a therapeutic virus. As a model system, we used an oncolytic recombinant vesicular stomatitis virus (rVSV). Replication kinetics were assessed by tissue culture infectious dose (TCID50), quantitative polymerase chain reaction, and optical cytometry. Transmission electron microscopy of high-pressure frozen, freeze-substituted samples was used to analyze the ultrastructure of rVSV-infected cells at different timepoints. Using this approach, we identified three key areas for bioprocess optimization. First, a decrease of the diameter of producer cells over the course of rVSV infection offers potential for online monitoring. Second, clusters of virions attached to the cell membrane of producer cells suggest the presence of cellular restriction factors, which could be overcome by strategies enhancing virus release. Finally, the observation of inclusion bodies surrounded by endoplasmic reticulum indicates a surplus of genomic copies within the producer cells, representing a bottleneck that could be targeted to increase particle assembly after genome replication.

**IMPORTANCE:**

New biopharmaceutical products like oncolytic viruses demand the development of a broad analytical panel. Besides establishing new assays to characterize the product, development efforts can benefit from transferring advanced analytical technologies from related fields. Here, we showcase possible tie-ins for electron microscopy, a well-established technique for visualizing virus-host-cell interactions, in the biopharmaceutical development of an oncolytic virus. We report (i) a diameter decrease in producer cells alongside a destabilization of the inner cell architecture; (ii) intracellular accumulations of virions, a possible target for further optimizing virus harvest strategies; and (iii) accumulation of surplus genetic material in structures inside the cell, that is not assembled into infectious virions. These three observations illustrate how advanced microscopy techniques can open the door for new approaches in bioprocess design.

## INTRODUCTION

The demand for new therapeutic approaches is continuously growing, opening the door for novel therapeutic agents like oncolytic viruses. Vesicular stomatitis virus (VSV) has emerged as a promising candidate for oncolytic virotherapy. For new therapeutic modalities, such as viral therapeutics, product characterization necessitates a broad spectrum of analytical tools. This includes the optimization of known assays as well as the transfer of established methods from related fields. Physical (i.e., genomic) and infectious titers are essential to evaluate process development outcomes of a virus product, as demonstrated for recombinant VSV (rVSV) (1). Recent advancements include the development of a semi-automated infectivity assay (2) and an assay using MOI-dependent infection kinetics (3). A method transfer has been performed for analytical ultracentrifugation (AUC) to determine virus size and density (4). Further insights on virus morphology and host cell interactions can be generated via electron microscopy, which is already broadly applied in virology (5, 6). Therefore, a transfer to process development poses a logical consequence for advanced product characterization (7, 8).

VSV is a rhabdovirus with an approximately 11 kb negative sense single-stranded RNA genome (9), that is naturally associated with vesicular stomatitis in livestock (10). Similar to the rabies virus, VSV forms enveloped, bullet-shaped virions with a length of approximately 180 nm. These virions consist of a ribonucleoprotein (RNP) arranged in a helical nucleocapsid, a matrix protein (M), a lipid envelope, and a glycoprotein (G) (11–13). Notably, VSV is uniquely sensitive to interferons, making it a natural oncolytic agent against interferon-deficient tumor cells (14).

Pseudotyping VSV with the glycoprotein of LCMV reduces the induction of neutralizing antibodies and mitigates wild-type VSV neurotropism, making it a suitable therapeutic virus (15, 16). rVSV variants are currently evaluated in phase-I clinical trials for the treatment of patients with solid, refractory, advanced, and metastatic tumors (17). To enhance the oncolytic properties of rVSVs, it has been combined with immunogenic cargos that stimulate the immune system in the tumor-microenvironment after treatment (15). The high interest in VSV-based viral products has also established it as an interesting model system for bioprocess development (18, 19).

rVSV characterization benefits from extensive research on other rhabdoviruses, which share a well-defined replication cycle. Virus production is initiated by transcription and replication of the viral genome in shielded intracellular compartments. Replication of the rhabdovirus genome occurs in so-called inclusion bodies (IBs), dense regions in the cytoplasm of infected cells (20–22). Unlike the replication organelles of positive strand RNA viruses, which are luminal vesicles (23, 24), there is accumulating evidence that rhabdovirus-induced IBs resemble non-enveloped organelles (22). However, electron microscopy (EM) studies of IBs often reveal the presence of membranes (20, 21, 25), making IBs a structure that is still not yet fully understood. After translation of viral proteins and genome replication, viral ribonucleoproteins are transported to the plasma membrane for virus assembly and ultimately form the characteristic bullet-shaped virions (26). Using transmission electron microscopy (TEM), we demonstrated that rVSV replication induces changes in infected cells similar to those observed in other rhabdoviruses. For this study, HEK293 cells were infected with rVSV at varying multiplicities of infection (MOIs), and viral titers were monitored over a 33-h period. To minimize common artifacts in EM sample preparation, we used high-pressure freezing, a technique that preserves ultrastructural details in a state that is close to the native configuration (27).

Our findings reveal that the first virus budding occurs 5 h after infection and that, regardless of the MOI used for primary infection, rVSV achieves the same final titer at 26 h post infection (hpi). Based on the combined analysis of replication kinetics and ultrastructural changes, we identified possible targets for bioprocess optimization, paving the way for enhanced production strategies.

## MATERIALS AND METHODS

### Cell culture

HEK293 (ThermoFisher, #11625019) suspension cell cultures (150 mL in 500 mL shake flasks, Corning) were inoculated at 5 × 10^5^ cells mL^-1^ in BalanCD medium (Fuji Film) and incubated at 37°C with 5% CO_2_ at 120 rpm, as previously described (1).

### Seed virus

This study has been conducted with a rVSV pseudotyped with the glycoprotein of lymphocytic choriomeningitis virus (16) carrying an immunomodulatory cargo (1). Propagation and characterization of rVSV for research purposes have been described previously (19, 28, 29). The seed virus had a titer of 2.14 × 10^9^ infectious particles per milliliter as determined via TCID_50_ performed with baby hamster kidney (BHK) cells (2).

### Replication kinetics

Thirty minutes after inoculation with 5 × 10^5^ cells mL^−1^, cultures were infected with rVSV at an MOI of 1, 0.1, or 0.0005. Samples of 1 mL were collected at different time points over a 33 h period. Samples were either directly stored at −80°C or treated with 200 mM sodium chloride to enhance virus release and subsequently centrifuged at 1,000 rpm for 5 min in reference to Gautam et al. (19). Centrifuge supernatants were stored at −80°C for subsequent analysis of extracellular genomic copies and infectious titer. The frozen aliquots were subsequently analyzed without further purification.

### Optical cytometry

Cell counts, viability, average cell diameter, and cell aggregation were analyzed using the NucleoCounter NC-202 (ChemoMetec) with Via2-cassettes. In brief, 60 µL of cell suspension was transferred to a Via2-cassette where 1.35 µL was analyzed by staining with acridine orange for total cell measurement and DAPI for dead cell count. The NC-View software (ChemoMetec) in version 2.1.0.28 (2023) was used for automated data analysis.

### Genomic titer

Upon thawing, RNA was extracted from unpurified samples using the MagMAX viral/pathogen nucleic acid isolation kit (Applied Biosystems) in combination with the KingFisher Flex (Thermo Fisher). Extra and intracellular RNA was made accessible by the kit’s binding solution, which contains Guanidinium thiocyanate. qPCR was then performed on extracted RNA using the Fast 1 step virus master mix (ThermoScientific) as described by reference 28, with the adjustment that 40 amplification cycles consisting of 3 s at 95°C for denaturation and 30 s at 60°C for extension were utilized.

### Infectious titer

Infectious titer was measured via TCID_50_ as described by Hochdorfer et al. (2). Briefly, BHK-21 cells were seeded in 96-well plates at 10,000 cells/well and infected with serially diluted samples after 24 h. Infected cells were incubated for another 96 h at 37°C with 5% CO_2_ and checked for cytopathic effects as signs of infection. Infected wells were counted, and the TCID_50_ was calculated using the Spearman-Kärber method (30, 31).

### Electron microscopy

For EM imaging, cells were cultured as described above. They were infected with rVSV at an MOI of 1. Samples of 875 µL were taken before infection and every 2 h after infection for a total of 10 consecutive hours. The samples shown in Fig. S3 were infected at MOI 0.0005 and chemically fixed 25 hpi. Samples shown in Fig. S3 were subjected to virus release treatment as described above. For every shown MOI, one shake flask was sampled. Infectious and genomic titer were measured to ensure comparability with the kinetic experiments. All samples for electron microscopy were chemically inactivated with 2% paraformaldehyde and 2.5% glutaraldehyde and subsequently stored at 2–8°C until further processing. For high-pressure freezing, samples were treated as described (32, 33) with the modification that suspension cells were frozen in sapphire-gold sandwiches instead of sapphire aluminum sandwiches. Carbon-coated, glow-discharged sapphire disks were treated with 0.01% Poly-L-Lysine (Pelco) for 20 min. Seven microliters of inactivated cell suspension was applied to each sapphire disk and incubated for 5 min. After high pressure freezing with a Wohlwend HPF Compact 01 (Engineering Office M. Wohlwend GmbH, Sennwald, Switzerland), samples were freeze-substituted following the protocol by Walther and Ziegler (34) using a substitution medium consisting of 0.1% uranyl acetate, 0.2% osmium tetroxide (OsO_4_), and 5% water, in acetone. The temperature was gradually elevated from −90°C to room temperature over 17 h. Samples were washed three times with 100% acetone and subsequently embedded in epoxy resin by treatment with ascending epoxy resin concentrations in acetone (1/3 epoxy resin for 1 h, 2/3 epoxy resin for 3 h, 100% epoxy resin overnight). Samples were left for 2 days at 60°C for polymerization. Seventy nanometer sections were cut with a Leica UCM ultramicrotome (Leica) equipped with a diamond knife (Diatome, Nidau, Switzerland). Sections were collected on formvar and carbon-coated, glow-discharged copper grids. TEM imaging was performed with a JEOL JEM 1400 electron microscope at 120 kV equipped with a Veleta CCD camera (Olympus). Representative images were selected from 50–100 images per sample.

## RESULTS

### rVSV infected cell cultures reach similar final titers independently of MOI

**Fig 1:**
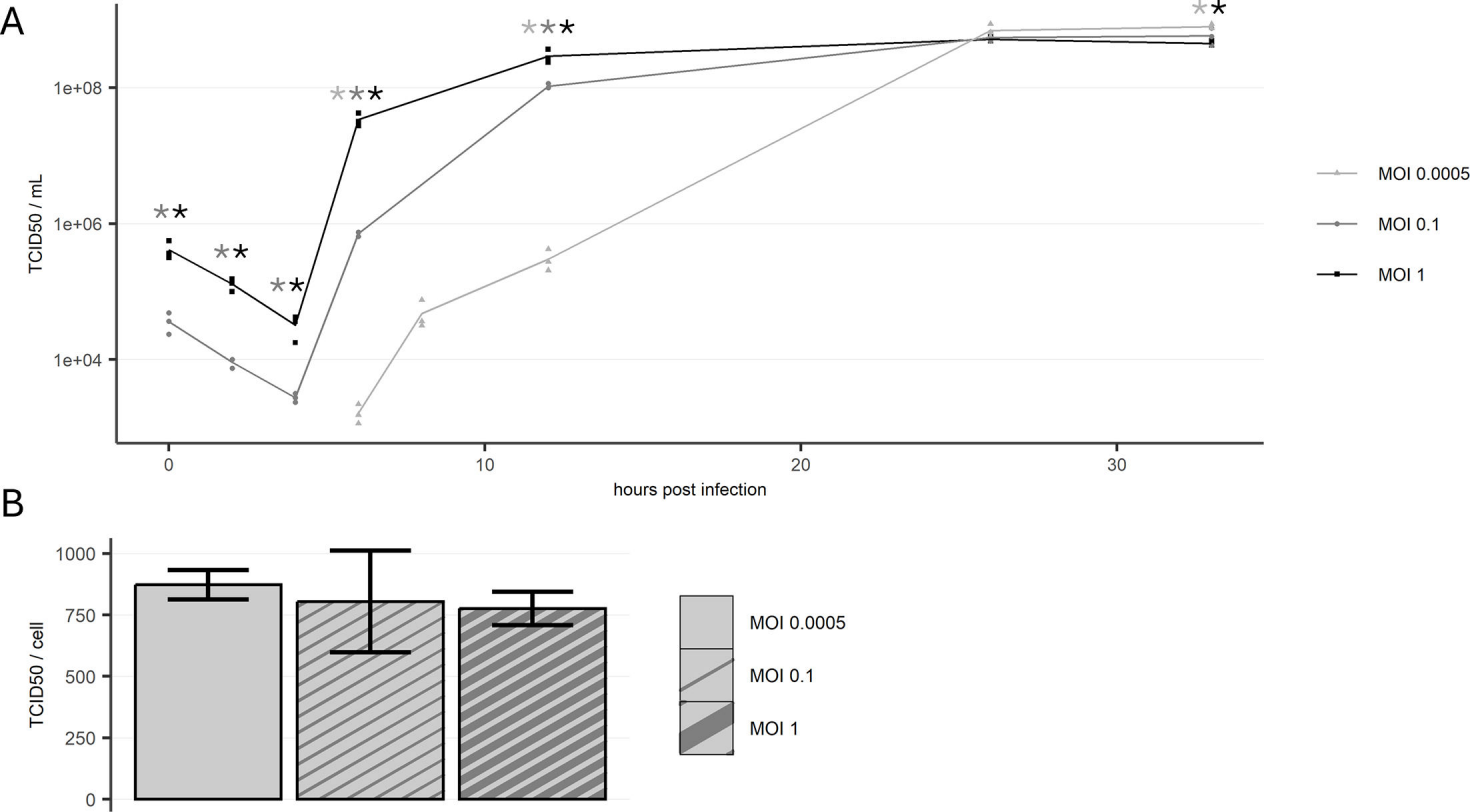
(**A**) Infectious titer of rVSV for cell cultures infected with three different MOIs. After an initial decrease, the infectious titer rises starting from 6 hpi before eventually reaching a maximum. The maximum titer reached in culture infected with MOI 0.0005 is significantly (*P* < 0.05) higher than in cultures infected with MOI 1. At any timepoint, significantly different data pairs are indicated by asterisks in the respective grayscale. (**B**) Cell- specific infectious virus yield for all three conditions shows no significant difference. *n* = 3 (biological replicates).

**Fig 2:**
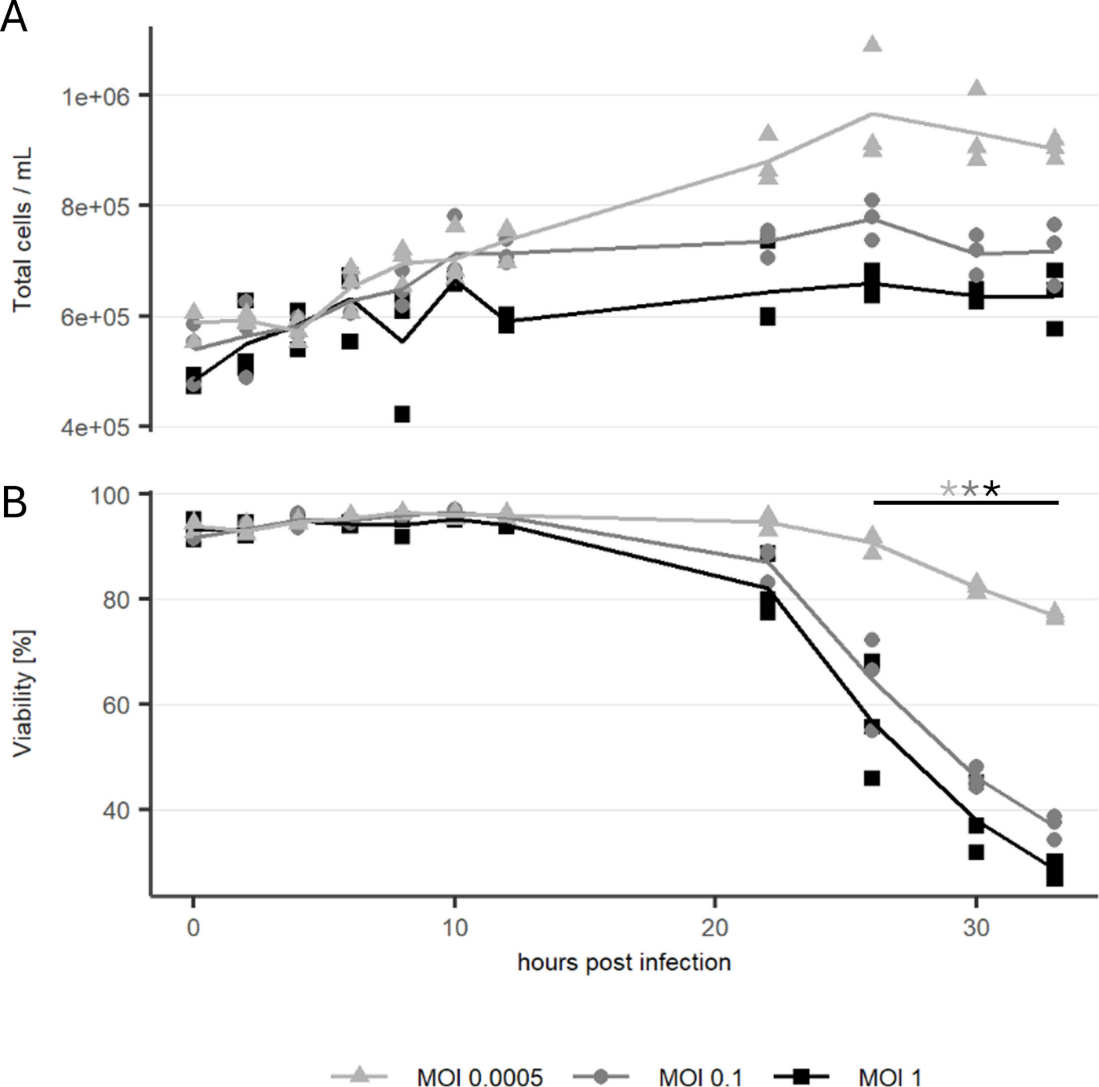
(**A**) Total cell count and (**B**) viability of cell cultures infected with rVSV at MOIs 0.0005, 0.1, and 1. *n* = 3 (biological replicates), asterisks mark significantly different viabilities between 26 hpi and 33 hpi (*P* < 0.05).

HEK293 cells were infected with MOIs of 1, 0.1, or 0.0005 and sampled at regular intervals to assess the replication kinetics of rVSV (Fig. 1A). Following an initial decline, the first increase in infectious titer was observed at 6 h post infection (hpi) for all MOIs. At 12 hpi, the difference in infectious titer between the tested MOIs reached its maximum. At this time, cultures infected with MOI 1 contained 3 × 10^8^ infectious particles/mL, which is close to the measured maximum of 5 × 10^8^ infectious particles/mL. In comparison, cultures infected at lower MOIs of 0.1 and 0.0005 produced titers of 1 × 10^8^ and 3 × 10^5^ infectious particles/mL, respectively. At 26 hpi, infectious particles/mL were the same in all cultures. Afterward, the infectious titer of MOI 1 began to decline, while cultures infected with the lowest MOI exhibited a continuous increase, ultimately reaching a maximum of 7.9 × 10^8^ infectious particles/mL at 33 hpi. Notably, cultures infected with this MOI were the only cultures showing an increasing cell number after 12 hpi. To evaluate the cell-specific virus yield, the TCID_50_/cell was calculated for the time of the maximum infectious titer (Fig. 1B). The results indicate that the cell-specific virus yields were not significantly different among the MOIs tested. While the total cell count (Fig. 2A) showed a slight increase for all MOIs, all cell viabilities declined significantly at the end of the experiment (33 hpi). Cultures with MOI 1 and 0.1 exhibited viabilities below 40%, whereas those infected at MOI 0.0005 maintained viability just under 80% (Fig. 2B).

### rVSV infection induces a diameter decrease in suspension cells

**Fig 3:**
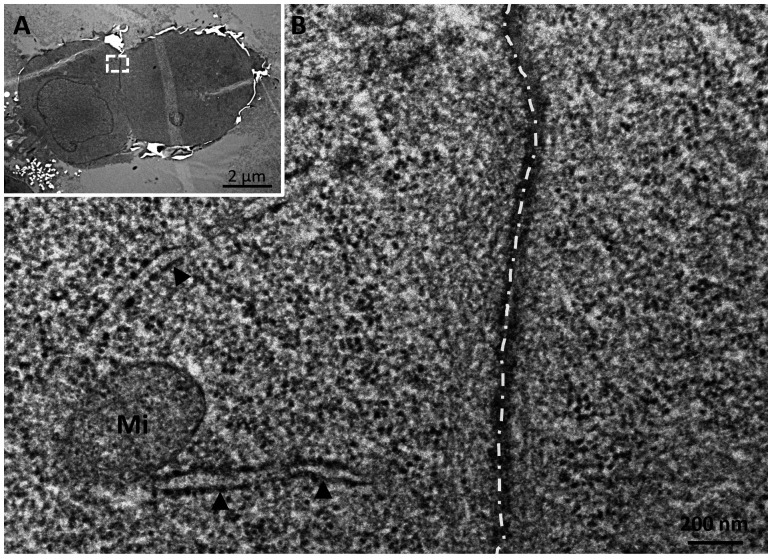
TEM imaging of uninfected, chemically fixed HEK293 suspension cells. (**A**) Overview. (**B**) Detail showing the area marked with the white rectangle in (**A**). Cells show mitochondria (Mi) and ER (black arrows), which forms contact sites with the mitochondria. Cell-cell contacts (dashed white line) appear. The figure shows representative results from one experiment. The scalebar represents 2 µm in (**A**) and 200 nm in (**B**).

Cells infected with MOI 1 were analyzed via EM imaging to focus on intracellular changes and virus budding throughout the first 10 h of infection. Since no reference studies on the ultrastructure of high-pressure frozen HEK293 suspension cells exist, we first characterized the ultrastructure of uninfected cells (Fig. 3A and B). Chemically fixed, high-pressure frozen, and freeze-substituted cells showed a good ultrastructural preservation with well visible phospholipid bilayers. Mitochondria showed visible cristae and contact sites with the endoplasmic reticulum (ER) (Fig. 3B). Also, cell-cell contacts were observed frequently (Fig. 3B).

**Fig 4:**
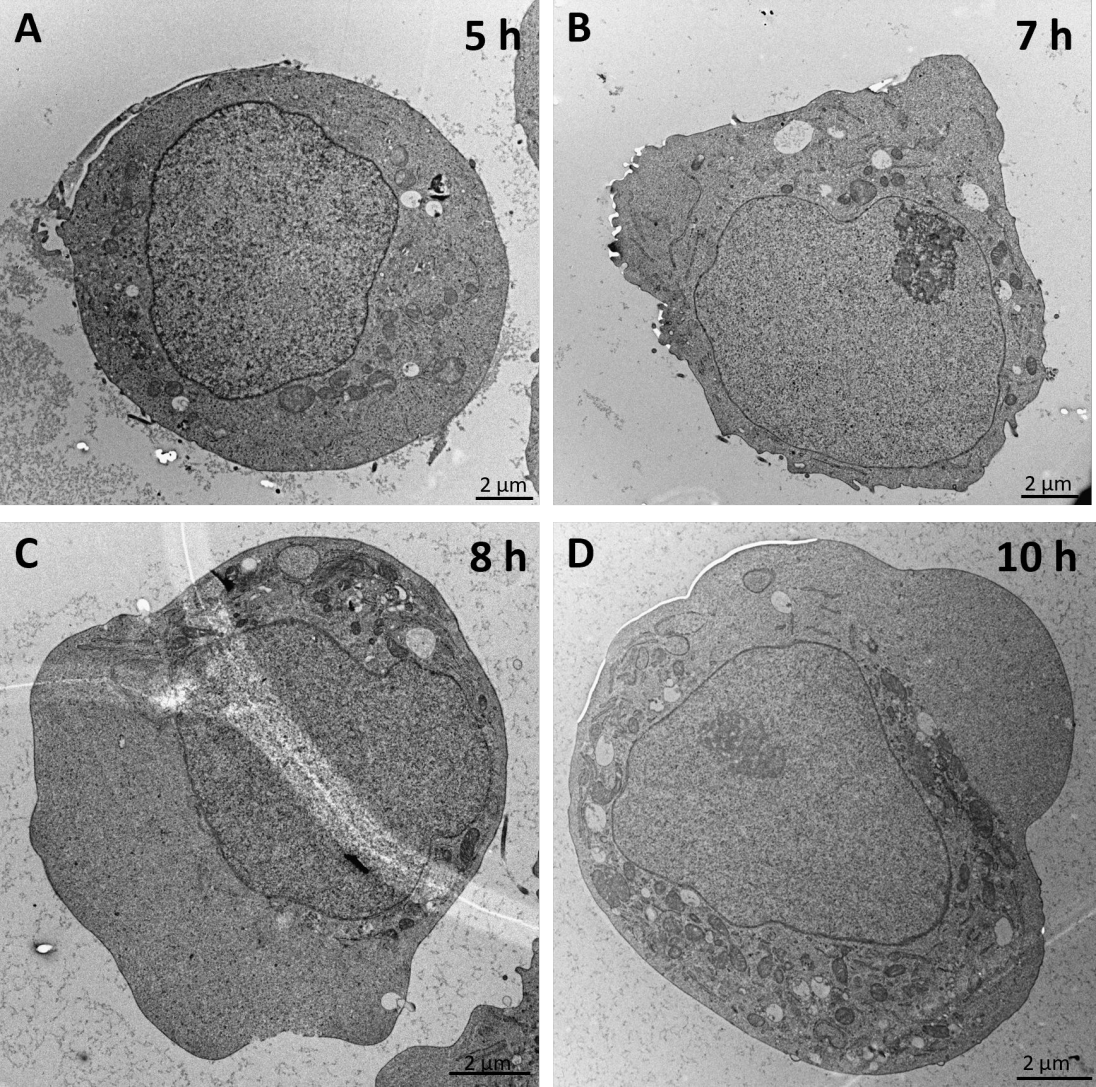
Morphological changes induced by rVSV infection after 5, 7, 8, and 10 h. Five hours after infection (**A**), the organelles are homogeneously distributed in the cytoplasm. Starting after 7 h (**B**) and extended after 8 h (**C**) and 10 h (**D**), the organelles are concentrated in the cytoplasm at one site of the cell. The figure shows representative results from one experiment. The scalebar represents 2 µm in each image.

**Fig 5:**
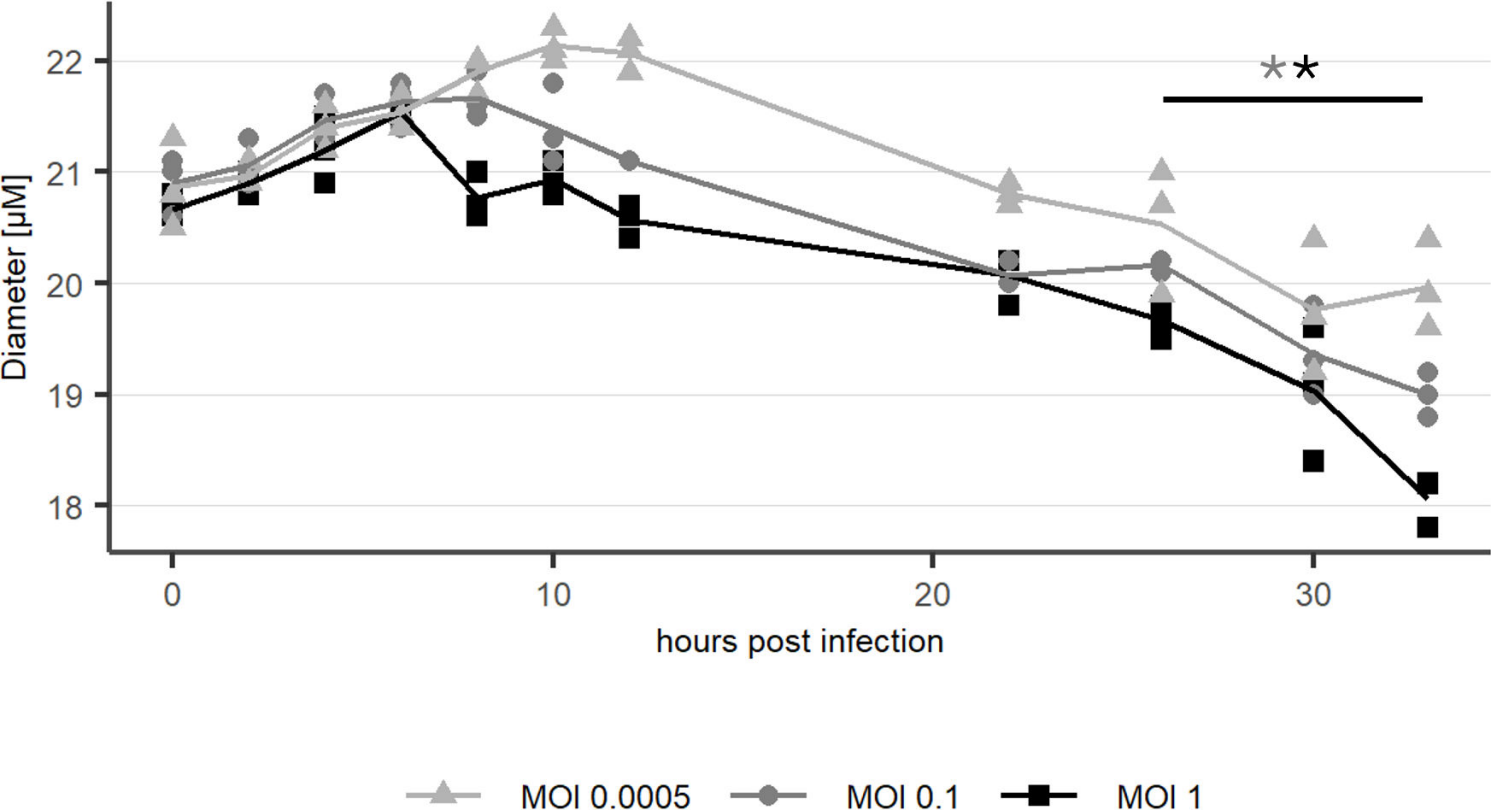
Optical cytometry: Average cell diameter in cell cultures infected with rVSV at MOI 0.0005, 0.1, and 1. *n* = 3 (biological replicates). Asterisks mark significantly different diameters between 26 hpi and 33 hpi (*P* < 0.05).

The cell morphology shows no observable changes during the early infection phase up to 5 hpi. Golgi apparatus, mitochondria, and ER show no signs of alteration in TEM images of samples fixed 2 hpi (data not shown), 5 hpi, and 7 hpi (Fig. 4A and B). Starting at 8 hpi, until 12 hpi, condensation of the cytoplasm around the nucleus was observed (Fig. 4C and D). Between 8 and 10 hpi, the average cell diameter as measured by optical cytometry starts to decrease significantly (*P* < 0.05) in cells infected with MOI 1 (Fig. 5), after an initial increase. In cultures infected by a lower MOI, especially at MOI 0.0005, only a fraction of the cells in the culture is infected this early, and thus, the decrease of average diameter is delayed. Until this timepoint, the average diameter increases for a longer time. For MOI 0.1, the decrease of average cell diameter was first observed 10 hpi, and for MOI 0.0005, a decreasing trend was visible starting at 12 hpi. The Augmented-Dickey-Fuller (ADF) test was performed with the outcome that none of the time series presented in Fig. 5 is stationary. Additionally, we observed no significant impact of rVSV infection on cell aggregation measured by optical cytometry (Fig. S1).

### Newly built rVSV virions are found in large clusters at the plasma membrane

**Fig 6:**
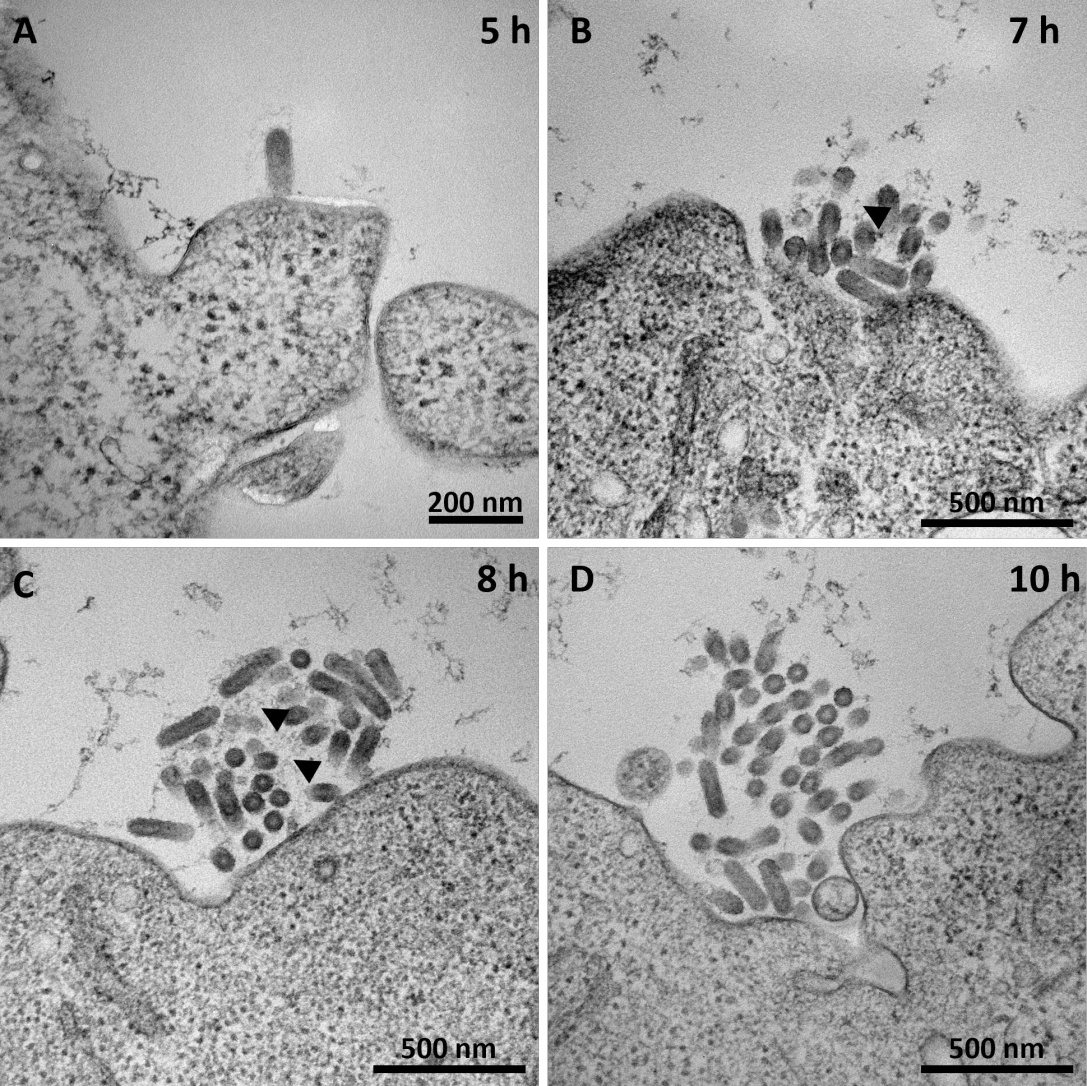
rVSV virions at the plasma membrane. Representative images of newly formed virions at (**A**) 5 h, (**B**) 7 h, (**C**) 8 h, and (**D**) 10 h after infection. Between the virions, diffuse material (black arrows) is visible. The figure shows representative results from one experiment. Scalebars represent 200 nm in (**A**) and 500 nm in (**B through D**).

In agreement with the data presented in Fig. 1, a first budding event of a bullet-shaped virion was visible 5 hpi (Fig. 6A). At the same time, vesicles start to bud from the plasma membrane (data not shown). At 6 hpi, single virions are visible at the cell membrane of infected cells (not shown). More rVSV virions are associated with the cell membrane in clusters of increasing size from 7 to 10 hpi (Fig. 6B through D), culminating to cells with several clusters at 10 hpi (Fig. S2). Due to different orientations of virions in the ultrathin section, the bullet shape is not always visible. Diffuse material with weak contrast is visible between virions (Fig. 6). Treatment of cells with a virus release agent led to a significantly higher titer than direct sampling without treatment (Fig. S3A). At the same time, no virion clusters are observed in TEM images of cells treated with a release agent. However, large intracellular accumulations of virions remain in place even after release (Fig. S3A and B).

### Surplus genomic copies accumulate in inclusion bodies in the cytoplasm of rVSV-infected cells

**Fig 7:**
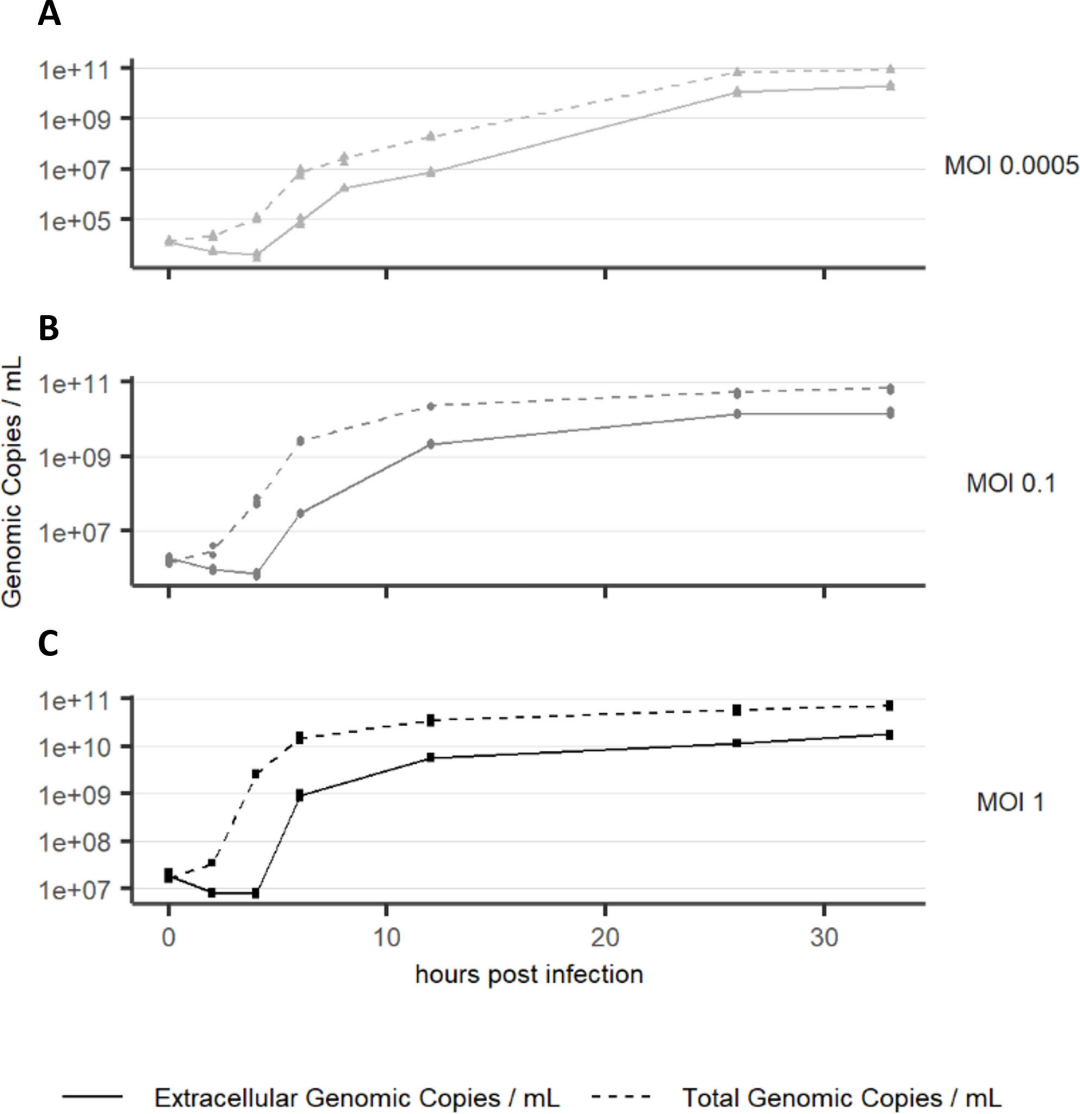
rVSV genomic copy concentration measured from host cell suspension (total genomic copies, dashed line) and centrifuge supernatant (extracellular genomic copies, solid line) after infection with (**A**) MOI 0.0005, (**B**) MOI 0.1, and (**C**) MOI 1.

**Fig 8:**
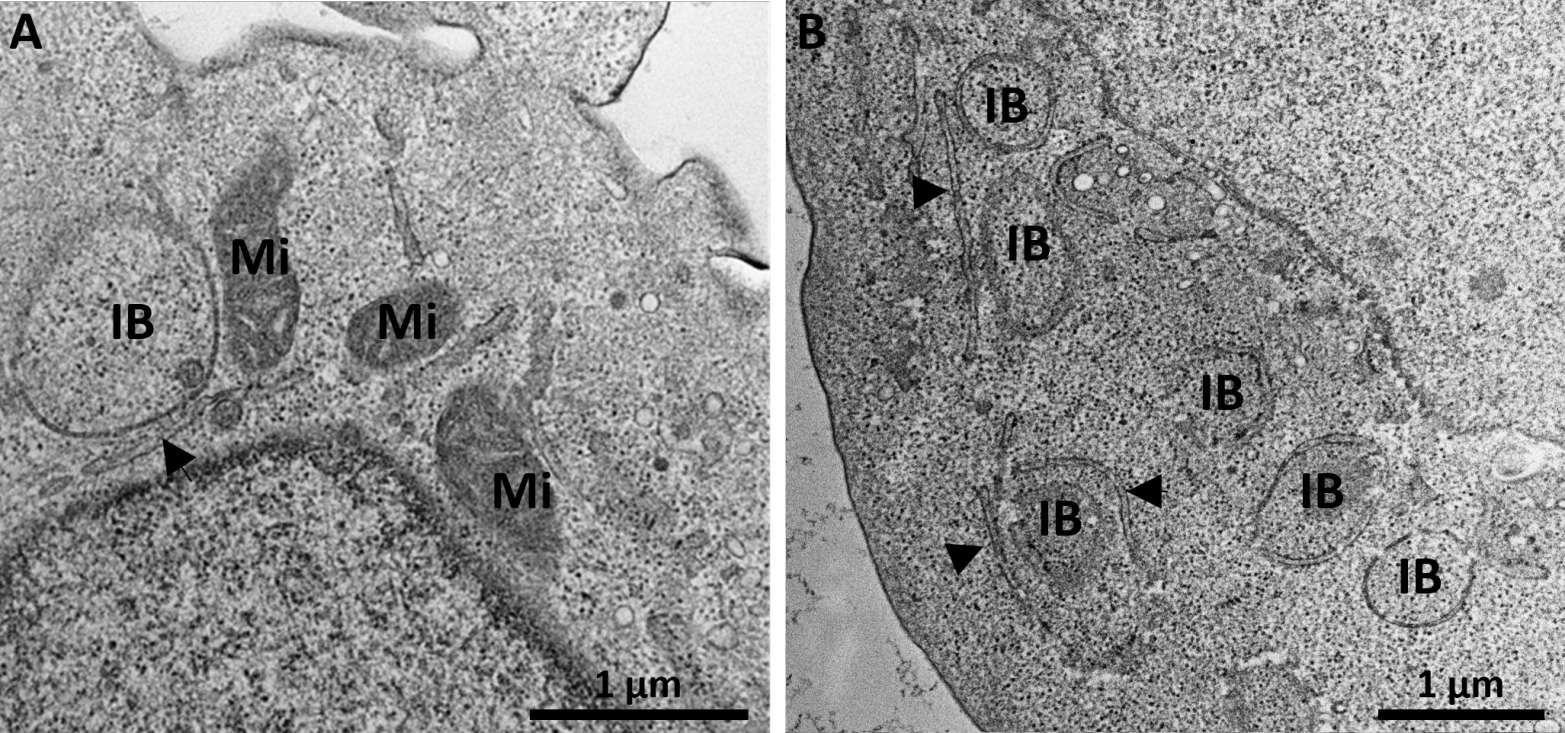
Inclusion bodies (IB) in rVSV-infected cells 8 hpi (**A**) and 10 hpi (**B**). ER (black arrows) is found in the vicinity of inclusion bodies. Mitochondria (Mi) show dilated cristae. The figure shows representative results from one experiment. The scalebars represent 1 µm.

Viral genomic titers were analyzed from samples including cells (total genomic titer) in addition to the cell culture supernatant (extracellular genomic titer), as displayed in Fig. 7. Extracellular genomic copies correspond to virus particles outside of the cell, while total genomic copies also include the rhabdovirus replication machinery inside the cells, like the IBs (Fig. 8). Extracellular genomic copies decrease for 4 h after infection before they start to increase. Total genomic copies already increase at 2 hpi. This delay between total and extracellular genomic titer is observed for all tested MOIs but is most pronounced at MOI 1. Total genomic copies reach a final titer which is approximately fivefold higher than extracellular genomic copies, indicating a significant number of genomic copies that remain inside the cells. At 8 hpi, well visible, single inclusion bodies were observed in the cytoplasm of infected cells (Fig. 8). All IBs observed in our study were associated with ER (Fig. 8) and were situated near the nucleus. At later timepoints, several IBs were observed at different locations in the cytoplasm of one cell (Fig. 8).

## DISCUSSION

The study demonstrates that higher MOIs result in faster virus replication kinetics. The detection of increase of infectious titer in cell culture supernatant occurred between 4 and 6 hpi, which is in alignment with the first budding of virions observed by electron microscopy at 5 hpi. As expected, lower MOIs resulted in a portion of the culture being uninfected, allowing continued cell growth and, thus, higher cell counts. Given the constant cell-specific virus yield (Fig. 1B), this led to a linear increase in overall viral titers. Thus, lower MOIs support a more productive bioprocess by increasing the density of producer cells. Higher MOIs, on the other hand, result in a more rapid production process. We attribute the fast replication kinetics of rVSV to its simple replication cycle, which is generally more rapid than replication of viruses with more complex replication cycles like retroviruses (35).

Ultrastructure data of high-pressure frozen mammalian suspension cell lines are scarce. In this study, ultrastructural preservation is consistent with prior research conducted on chemically pre-fixed, high-pressure frozen cells (36), providing clear visibility of the phospholipid bilayers. Frequent cell-cell contacts were observed in uninfected cells, which is consistent with reports of upregulated cell-adhesion associated genes in suspension cells (37). However, rVSV infection did not influence cell aggregation, contrary to virus-induced alterations reported in other systems (38).

Virus-induced membrane remodeling was less pronounced than that observed for infection with positive-strand RNA viruses such as Zika virus (39) or SARS-CoV2 (40). In later infection stages, starting from 8 hpi, condensation of cell organelles near the nucleus was observed, and the average cell diameter decreased. In combination with condensation of the cytoplasm around the nucleus, this suggests cytoskeletal damage, a phenomenon linked to cell rounding in adherent cultures (41). Such rounding has been employed as a robust infection indicator for rVSV and other viruses (3). A connection between viral infection and cell diameter has been reported previously for the baculovirus-insect-cell expression system (42). However, the authors correlated an increase in diameter with the productivity of protein production in cell culture (42). The diameter reduction in our case could serve as a potential parameter for determining the point of full culture infection, a metric relevant to industrial bioprocesses.

rVSV virions were found in clusters close by the plasma membrane, demonstrating an ongoing, but not completed virus budding and release process. Over time, these clusters increased in size correlating with an increased infectious titer in the supernatant. We attribute this observation to the presence of cellular restriction factors that cause newly built virions to stick to the cell surface, as observed in other systems, such as for SARS-CoV-2-infected cells (27). Virus release treatment leads to a higher titer in the supernatant. The presence of large intracellular virion accumulations after the release step indicates that established release strategies target extracellular virion clusters rather than intracellular virions.

The most prominent virus-induced alteration in the cytoplasm of infected cells was the formation of inclusion bodies, consistent with previous reports of genome replication sites for VSV. While IBs were shown to have the characteristics of liquid organelles (22), we found that IBs were associated with rough endoplasmic reticulum, in agreement with previous observations for rhabdoviruses (20, 21, 25). Notably, budding from ER-derived membranes next to IBs, as observed for lyssaviruses (43, 44), was absent in rVSV-infected cells, suggesting that virion morphogenesis is limited to the plasma membrane in the timeframe observed in this study. About 80% of the genomic copies remained within the cells. With an increasing number of total genomic copies, the abundance of IBs also increased at 10 hpi. This observation suggests a bottleneck between genome replication and budding of new virions, leading to the accumulation of surplus genomic copies in IBs. Notably, this bottleneck in rVSV replication is different from the retention of assembled virions in the endoplasmic reticulum, as observed in Zika virus-infected glioblastoma cells (36).

### Conclusion

The presented study identifies different targets for bioprocess optimization on the example of rVSV production. In summary:

- While higher MOIs reach their respective maximum faster, lower MOIs can reach a significantly higher titer (*P* < 0.05). Thereby, cell-specific virus yield is independent of the initial MOI. Here, it was 820 ±50 TCID_50_/cell.
- The average diameter of cells decreases over the course of infection, hinting at cytoskeletal destabilization. This can potentially be used to monitor rVSV progression of infection in the cell culture by a simple, easy-to-use cell counting method.
- rVSV virions remain associated with the cells in large clusters after budding from the plasma membrane, pointing out a primary target for virus release agents. Intracellular virions, however, could pose a potential secondary target for novel release agents.
- Surplus genomic copies accumulate in inclusion bodies which are partially enclosed by rough endoplasmic reticulum. Focusing on the relation between IBs, ER, and the transition from rVSV RNPs to budding virions can be targeted to optimize virus production.

## SUPPLEMENTAL MATERIAL

Figure S1 spectrum.01063-25-s0001.tif Aggregates measured by optical cytometry.

Figure S2 spectrum.01063-25-s0002.tif Overview image of an infected cell.

Figure S3 spectrum.01063-25-s0003.tif Visualization of rVSV release.

Supplemental material spectrum.01063-25-s0004.docx Legends for Figures S1 to S3.

ASM does not own the copyrights to Supplemental Material that may be linked to, or accessed through, an article. The authors have granted ASM a non-exclusive, world-wide license to publish the Supplemental Material files. Please contact the corresponding author directly for reuse.
