# Supplementary figures and images for: Integrating replication kinetics and ultrastructural analysis to identify targets for optimizing rVSV bioproduction
Source: Microbiol Spectr. 2026 Mar 24;14(5):e01063-25. doi: 10.1128/spectrum.01063-25 (PMC13141878; doi:10.1128/spectrum.01063-25)

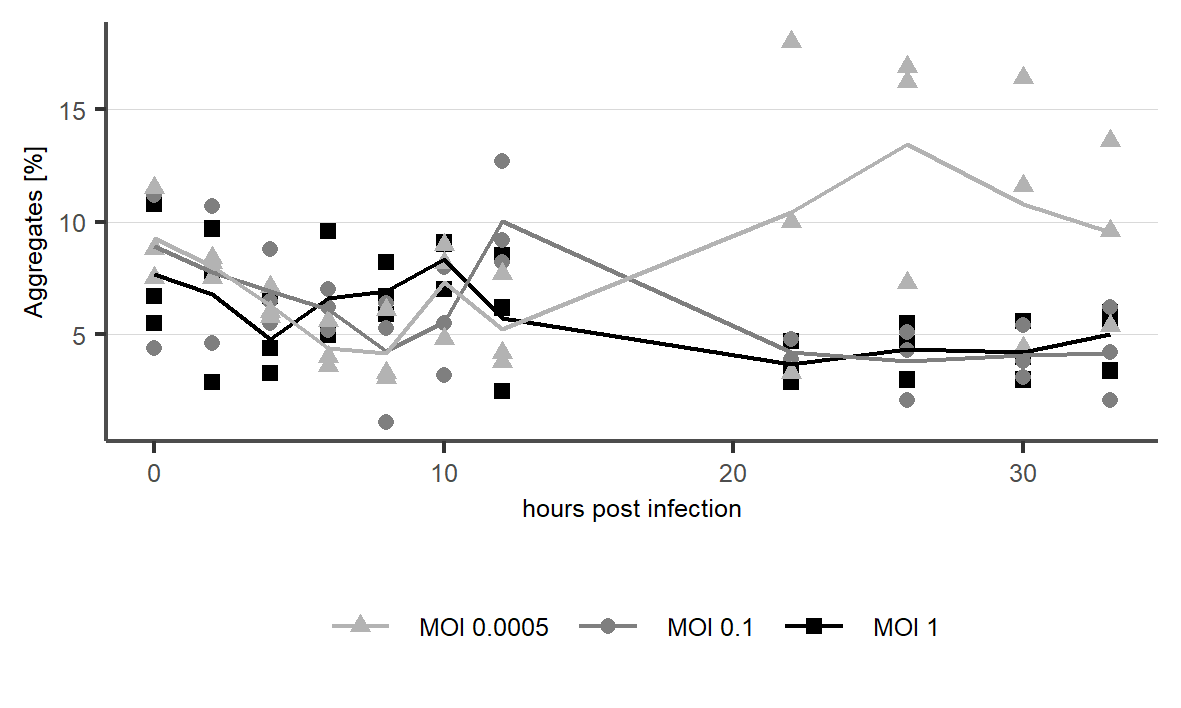

Supplement: Figure S1 — Aggregates measured by optical cytometry. [file spectrum.01063-25-s0001.tif]

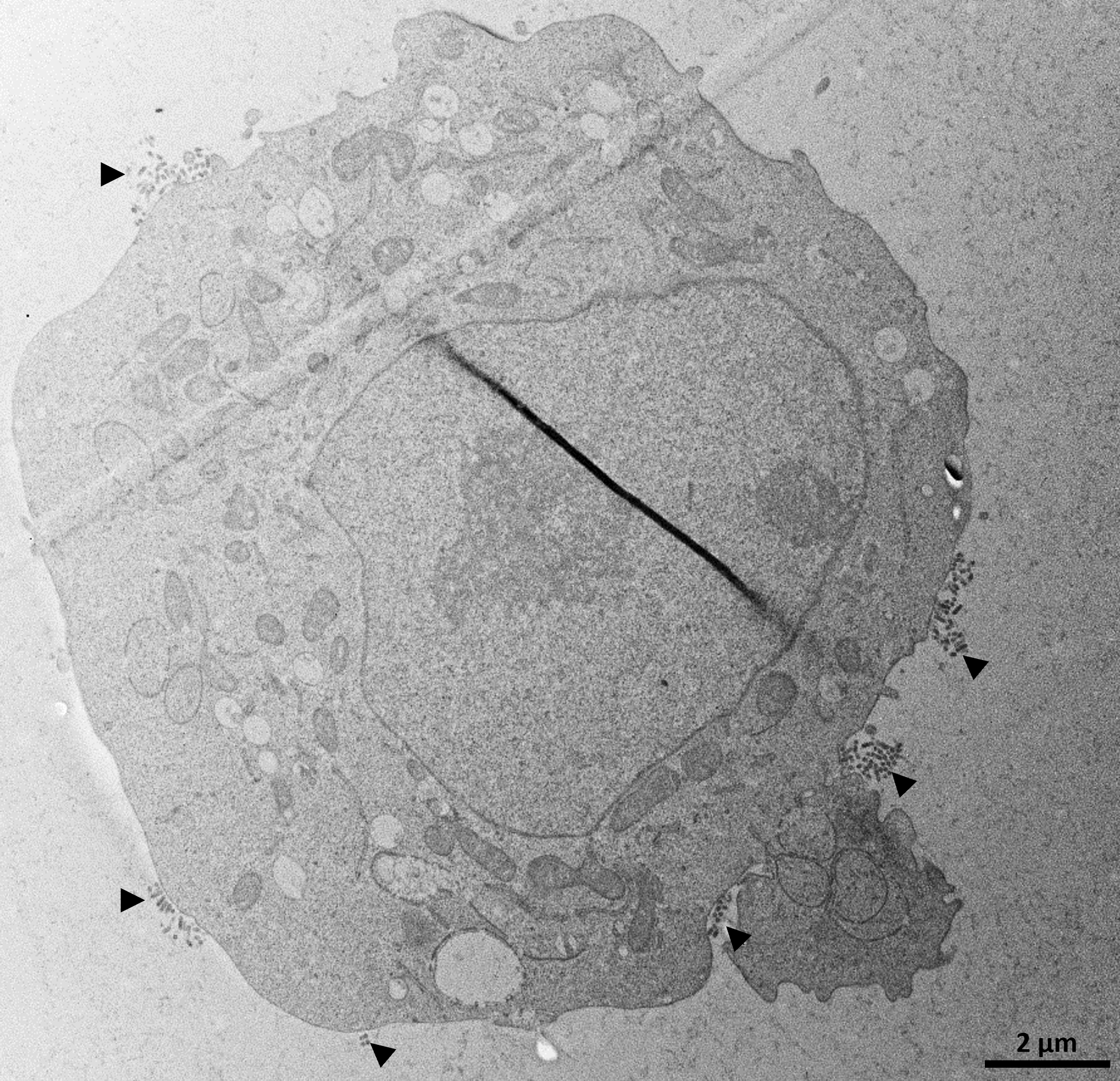

Supplement: Figure S2 — Overview image of an infected cell. [file spectrum.01063-25-s0002.tif]

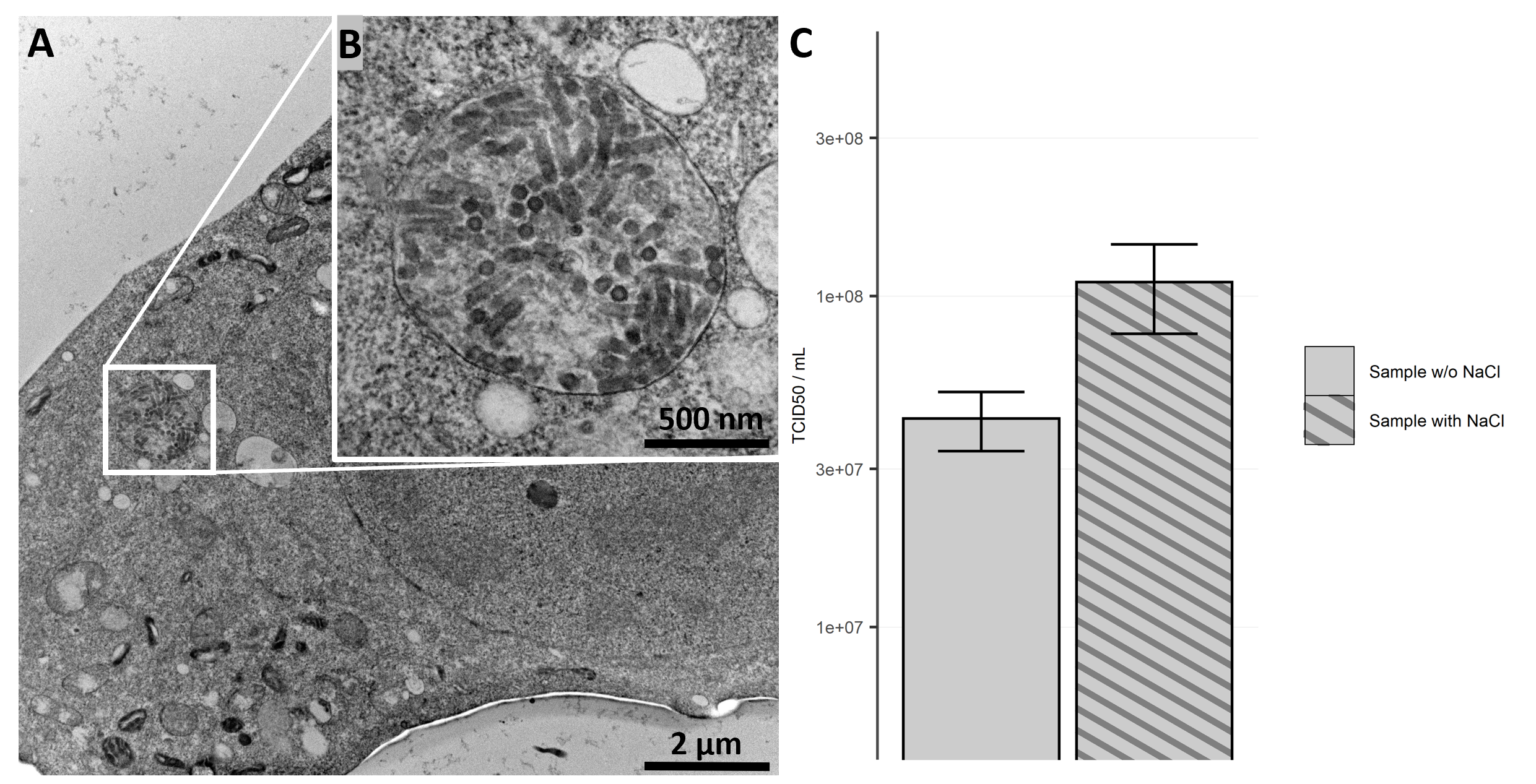

Supplement: Figure S3 — Visualization of rVSV release. [file spectrum.01063-25-s0003.tif]
