# Supplementary material for: Integrating replication kinetics and ultrastructural analysis to identify targets for optimizing rVSV bioproduction
Source: Microbiol Spectr. 2026 Mar 24;14(5):e01063-25. doi: 10.1128/spectrum.01063-25 (PMC13141878; doi:10.1128/spectrum.01063-25)
Supplement: Supplemental material — Legends for Figures S1 to S3. [file spectrum.01063-25-s0004.docx]

**Supplementary Information:**

*Supplement Figure 1: Cell aggregation as determined by optical cytometry.*

*Supplement Figure 2: Overview of a rVSV-infected cell 10 hpi showing several extracellular clusters of bullet shaped virions (black arrows) near the cellular membrane. The scalebar represents 5 µm.*

*Supplement Figure 3: (A) Overview of a cell treated for virus release and (B) detail showing large vesicles filled with virions that are not released from inside the cell. (C) Infectious titer as determined without release step (light grey) and with release step (striped) shows the benefit of performing a dedicated release step.*
